# Complete Genome Sequence of Temperature-Dependent Pigment-Producing Serratia marcescens ATCC 274

**DOI:** 10.1128/MRA.00164-20

**Published:** 2020-04-09

**Authors:** Suzuka Yabe, Jun Fukushima

**Affiliations:** a Department of Biotechnology, Akita Prefectural University, Akita City, Japan; b Biotechnology Center, Akita Prefectural University, Akita City, Japan; Indiana University, Bloomington

## Abstract

Serratia marcescens ATCC 274 produces the red pigment prodigiosin and the biosurfactant serrawettin W1 at 30°C but not at 37°C. A complete, high-quality genome sequence of S. marcescens ATCC 274 was obtained and found to comprise a single 5,148,533-bp circular genome with 4,799 genes.

## ANNOUNCEMENT

Serratia marcescens is a facultative anaerobic Gram-negative bacillus belonging to the *Enterobacteriaceae* and is an opportunistic infection-causing bacterium (1). S. marcescens is generally known for its production of secondary metabolites, such as the red pigment prodigiosin (2) and the biosurfactant serrawettin W1 (3). S. marcescens ATCC 274, which was subjected to genome analysis in this study, was previously shown to produce prodigiosin and serrawettin W1 at 30°C but not at 37°C (4). S. marcescens ATCC 274 has been extensively studied for its temperature-dependent production of secondary metabolites (5). However, the mechanism of this temperature-dependent regulation has yet to be clearly elucidated. Here, we report on the results of S. marcescens ATCC 274 genome analysis as a basis to elucidate the mechanisms of its temperature-dependent secondary metabolite production.

S. marcescens ATCC 274 was obtained from the American Type Culture Collection and was cultured aerobically for 16 h at 26°C in Difco nutrient broth. Genomic DNA (gDNA) was extracted using the surfactant cetyltrimethylammonium bromide and was purified using a NucleoSpin gDNA clean-up column (Macherey-Nagel, Germany). One microgram of gDNA was used for long-read library preparation with a ligation sequencing kit (catalog number SQK-LSK108; Oxford Nanopore Technologies, UK). Long-read sequence reads (171,374 reads with an average read length of 3,319 bp) were obtained with the GridION X5 platform (Nanopore). Raw sequence data were base called using Guppy v.3.0.3 software. Short (<1,000-bp) and/or low-quality (<Q10) reads were filtered using NanoFilt v.2.5.0 software (6), resulting in a total of 56,492 reads with an average read length of 7,377 bp.

Five hundred nanograms of the same genomic DNA was used for the Illumina library and was enzymatically fragmented to produce 300- to 350-bp-long DNA fragments using the Nextera DNA Flex library preparation kit (Illumina, CA) according to the manufacturer’s instructions. Paired-end (2 × 150-bp) reads (3,550,000 reads with an average read length of 153 bp) were obtained with the MiSeq system (Illumina), and raw reads were filtered (<10 bp and/or <Q30) using fastp v.0.14.1 software (7), resulting in a total of 3,198,000 reads with an average read length of 148 bp. The genome sequence of S. marcescens ATCC 274 was assembled by combining long-read and short-read sequences using the Unicycler v.0.4.4 pipeline (8) and visualized using Bandage v.0.8.1 software (9). The contig sequence obtained was polished three times using Pilon v.1.14 software (10) until errors were not detected; finally, 29 regions were corrected. All of the computer programs were used with their default settings. The final complete genome consisted of 5,148,533 bp, with a G+C content of 59.5% and 105× coverage. Automatic annotation using DFAST v.1.1.4 (11) resulted in 4,799 coding sequences, with 22 rRNA genes and 96 tRNA genes detected. Further analysis showed that the *pig* gene cluster and the *pswP* gene, both of which are required for the production of prodigiosin, were present. Notably, four *luxR* homologues were found. These homologues are considered unlikely to be involved in acyl-homoserine lactone-based quorum sensing, because there are no *luxI* homologues in this genome.

### Data availability.

The complete genome sequence of S. marcescens ATCC 274 has been deposited in DDBJ/EMBL/GenBank under accession number AP021873. The BioProject accession number is PRJDB8907, and the SRA accession numbers are DRR196107 (Illumina) and DRR196108 (Nanopore).
